# Ejaculatory abstinence duration impacts semen parameters: Insights from a retrospective analysis in male infertility on 23,527 analyses

**DOI:** 10.3389/fendo.2025.1529262

**Published:** 2025-02-10

**Authors:** Giorgio Ivan Russo, Maria Giovanna Asmundo, Andrea Cocci, Ali Saber Abdelhameed, Annalisa Liprino, Filippo Giacone, Debora Lombardo, Antonino Guglielmino, Sandrine Chamayou

**Affiliations:** ^1^ Urology Section, University of Catania, Catania, Italy; ^2^ Urology Section, University of Florence, Florence, Italy; ^3^ Department of Pharmaceutical Chemistry, College of Pharmacy, King Saud University, Riyadh, Saudi Arabia; ^4^ Unità di Medicina della Riproduzione, Centro HERA, Catania, Italy

**Keywords:** sperm, infertility, age, male, sperm analysis

## Abstract

**Purpose:**

Ejaculatory abstinence (EA) duration is recognized to impact semen parameters. This study aims to evaluate the effects of varying EA durations on semen quality parameters, distinguishing between normospermic and sub-fertile men, and to provide insights into tailored abstinence recommendations for improved fertility outcomes.

**Methods:**

We retrospectively analyzed 23,527 semen samples from men undergoing infertility evaluation from 2013 to 2024. Semen parameters, including sperm concentration, motility, and morphology, were assessed post-abstinence (2–7 days) according to WHO guidelines. Group differences were analyzed, focusing on sperm parameters across abstinence periods in normospermic versus patients with sperm abnormalities.

**Results:**

In normospermic patients we found a trend increase from day 1 to day 7 of abstinence time regarding total sperm count (million) (92.4 vs. 191.1; p<0.01), sperm concentration (million/ml) (44.5 vs. 72.0; p<0.01) and morphology (6 vs. 12.5; p= 0.03) but not regarding motility (A+B) (50.0% vs. 48.0%; p=0.43). Conversely, in the population of patients with sperm abnormality, we found a significant trend increase from day 1 to day 7 of TSC (16.38 vs. 56.0; p<0.01), sperm concentration (million/ml) (8.0 vs. 18.0; p<0.01) and morphology (3.0 vs. 5.0; p<0.01). Interestingly, we found a significant trend decrease of motility (A+B) (28.0% vs. 21.0%; p<0.01) and pH (8.1 vs. 7.9; p<0.01) In patients affected by asthenospermia, motility (A+B) dropped significantly from day 1 to day 7 (11.8% vs. 6.1%; p<0.01) and also in patients with teratospermia morphology dropped significantly (2.13% vs. 1.26%; p<0.01).

**Conclusion:**

The findings support the use of tailored abstinence guidelines to optimize semen quality based on patient-specific semen profiles, with normospermic men benefiting from longer abstinence durations to increase concentrations, while patients with motility or morphology impairments, may benefit from shorter abstinence periods to mitigate sperm quality declines.

## Introduction

Infertility is a substantial challenge affecting approximately between 48.5 and 72.4 million couples (1). Within this landscape, male factors contribute to half of all infertility cases, underscoring the need for focused research and targeted interventions. Despite notable progress in Assisted Reproductive Technology (ART), the evaluation of male infertility often lacks the thorough attention afforded to female reproductive health, potentially limiting the success of ART procedures. A more comprehensive approach to assessing male fertility is essential to optimize clinical outcomes and improve the likelihood of successful conception.

Semen analysis is currently the primary diagnostic tool for identifying male factor infertility. This analysis examines several sperm parameters, including concentration, motility, and morphology, which are critical indicators of male reproductive health. One of the factors that significantly impacts these sperm parameters is the duration of ejaculatory abstinence (EA) before sample collection (2). This period of abstinence can influence the quality and quantity of sperm present in a sample, potentially affecting the results of semen analysis and subsequent clinical decisions (3–6).

Guidelines from major health organizations differ slightly on the recommended duration of EA. The World Health Organization (WHO) advises an abstinence period of 2–7 days, while the European Society of Human Reproduction and Embryology (ESHRE) narrows this range to 3–4 days (7, 8). However, these recommendations are based on data from the general population and may not address the specific needs of men with infertility issues. Research suggests that individualized EA recommendations could be beneficial, as different abstinence periods may optimize semen quality in unique ways for those experiencing infertility (9, 10).

The aim of the present study is to understand the influence of EA on semen parameters in patients with normal sperm parameters and sperm abnormalities.

## Patients and methods

We retrospectively analyzed sperm analysis results performed in a single institution for infertility work up from 2013 to 2024, data regarding the ejaculatory abstinence of every patient has been reported. Our study included patients aged 18 years seeking for fertility. We collected data on each patient’s age and conducted a physical exam, recording measurements for height, weight, and BMI. The study protocol received Institutional Review Board approval from Centro HERA - UMR (Approval No. 1/2023), and all participants provided informed consent upon enrollment.

Data were cross-verified and only records meeting predefined criteria for reliability were included. Patient-reported events were triangulated with other objective data like laboratory results to ensure that samples were collected correctly.

Semen samples were obtained through masturbation into a sterile container after a period of sexual abstinence lasting 2–7 days. Analyses were conducted immediately post-liquefaction and evaluated for seminal volume, sperm concentration, progressive motility, and morphology according to the 5h and 6th WHO guidelines (11, 12).

Both external quality control (EQC) and internal quality control (IQC) were conducted in accordance with the 2010 WHO guidelines (12), confirming no significant differences between the two analyses. EQC was performed biannually, comparing sample analyses from our laboratory with those performed by the Quality Control Team of the Central Laboratory at the University Teaching Hospital Policlinico “G. Rodolico-San Marco.” For IQC, biannual replicate measurements were taken from separate aliquots of semen samples by two technicians in our Seminology Laboratory. Analyses were performed manually by two dedicated biologists. Warning and action control limits were determined using an S-chart plot 30, and calibration of pipettes, counting chambers, and other equipment assessments were completed annually (12).

Oligozoospermia has been defined as < 39 million or < 15 million spermatozoa per ml; asthenospermia, as motility A+B < 32% and teratozoospermia, defined as normal morphology < 4%. Oligoasthenoteratozoospermia (OAT), defined by the presence of all these abnormalities.

Patients with oligoasthenoteratospermia (OAT) or isolated oligospermia, asthenospermia and teratospermia have been classified such “sperm abnormal”, patients with no semen alterations have been classified such “normospermic”.

Normally distributed continuous variables were reported as median (interquartile range, IQR), and group differences were tested using Student’s t-test or the Mann–Whitney U-test, based on the distribution (tested with the Kolmogorov–Smirnov test). A not-parametric trend analysis has been performed in normospermic and sperm abnormal groups by abstinence days in order to reveal the eventually relation between abstinence time and semen parameters variations.

EA has been analyzed in tertiles, with the lowest quartile (Q1) as the reference group. Linear regression analysis has been used to test association between continuous variables.

Logistic regression models to assess the relationship between EA categories and OAT as categorical variable, adjusted for potential confounders, including age, BMI and smoking status. All statistical analyses were performed using Stata (Stata Statistical Software: College Station, TX: Stata Corp LP), with statistical significance set at p < 0.05.

## Results

A total of 23,527 sperm analysis have been collected, 3,338 normospermic and 20,139 with sperm abnormality.

**Table 1** provides an overview of the clinical characteristics of the cohort under study. The median age of participants is 43 years, with an interquartile range (IQR) from 38 to 48 years. The median body mass index (BMI) is 26.2 kg/m², falling within the overweight range, with an IQR from 24.2 to 29.1. The median abstinence time prior to semen collection is 4 days, with a narrower IQR of 3 to 4 days.

**Table 1:** Clinical characteristics of the cohort.

Variables	
Age (years), median (IQR)	43.0 (38.0 – 48.0)
BMI (kg/m^2^), median (IQR)	26.2 (24.2 – 29.1)
Abstinence time (days), median (IQR)	4.0 (3.0-4.0)
Total sperm count (million), median (IQR)	41.58 (8.0-116.0)
Sperm concentration (million/ml), median (IQR)	17.1 (3.0-44.0)
Motility A+B (%), median (IQR)	29.0 (17.0-40.0)
Motility C (%), median (IQR)	29.0 (17.0-40.0)
Motility D (%), median (IQR)	24.0 (15.0-34.0)
Morphology (%), median (IQR)	5.0 (3.0-9.0)
pH, median (IQR)	8.1 (7.9-8.3)

IQR, interquartile range; BMI, body mass index.

**Table 2** compares the clinical characteristics between normospermic and sub-fertile patients. Notable differences include a significantly lower sperm concentration and total sperm count in sub-fertile patients, with median values of 12.1 million/ml and 29.7 million, respectively, compared to 48.0 million/ml and 131.3 million in normospermic patients (p < 0.01). Motility (A+B), representing progressive movement, is also much lower in sub-fertile patients at 26%, compared to 50% in normospermic individuals. Additionally, sub-fertile patients display a higher percentage of immotile sperm (Motility D) and abnormal morphology, with only 5% of sperm showing normal form versus 11% in normospermic patients. Other parameters, such as abstinence time, pH, and leukocyte levels, also differ significantly between the two groups, underscoring the varied semen quality in sub-fertile versus normospermic men.

**Table 2:** Clinical characteristics of the cohort in normospermic and sperm abnormal patients.

Variables, median (IQR)	Normospermic (N = 3,388)	Sperm abnormal (N = 20,139)	p-value
Age (years)	44.0 (44.0-49.0)	43.0 (38.0-48.0)	<0.01
BMI (kg/m^2^)	26.1 (24.1-28.9)	26.2 (24.2-29.1)	0.18
Abstinence time (days)	3.0 (3.0-4.0)	4.0 (3.0-4.0)	<0.01
Sperm concentration (million/ml)	48.0 (32.5-76.0)	12.1 (2.25-35.75)	<0.01
Total sperm count (million)	131.28 (84.0-202.9	29.7 (5.8-92.4)	<0.01
Sperm volume (ml)	2.8 (2.0-3.7)	2.8 (1.97-4.0)	0.84
Motility (A+B)(%)	50.0 (45.0-58.0)	26.0 (15.0-35.0)	<0.01
Motility C (%)	16.0 (11.0-21.0)	25.0 (17.0-35.0)	<0.01
Motility D (%)	32.0 (26.0-39.0)	45.0 (35.0-55.0)	<0.01
Morphology (%)	11.0 (7.0-27.0)	5.0 (3.0-8.0)	<0.01
pH	8.0 (7.9-8.20)	8.1 (7.9-8.3)	<0.01
Leukospermia	0.1 (0.0-0.4)	0.25 (0.01-0.70)	<0.01

IQR, interquartile range; BMI, body mass index.

In normospermic patients we found a trend increase from day 1 to day 7 of abstinence time regarding total sperm count (TSC) (million) (92.4 vs. 191.1; p<0.01), sperm concentration (million/ml) (44.5 vs. 72.0; p<0.01) and morphology (6 vs. 12.5; p= 0.03) but not regarding motility (A+B) (50.0% vs. 48.0%; p=0.43) (**Table 3**).

**Table 3:** Sperm parameters according to ejaculation abstinence (EA) in normospermic patients.

EA (days)	TSC (million)	Concentration (million/ml)	Volume (ml)	pH	Motility A+B (%)	Motility C (%)	Motility D (%)	Morphology (%)
1 (n = 46)	92.4 (60.0-121.6)	44.5 (32-55)	2.3 (1.8-3.0)	8.1 (8.1-8.3)	50 (47-54)	17.5 (11-20)	32.5 (30-37)	6 (6-6)
2 (n = 321)	103.2 (72-140.8)	44 (32-64)	2.4 (1.8-3.0)	8.1 (7.9-8.3)	49 (45-55)	17 (11-21)	33 (29-40)	8 (5-10)
3 (n = 453)	122.4 (81.2-180)	48 (33-75)	2.8 (2-3.5)	8.1 (7.9-8.3)	49 (45-56)	19 (1-23)	31 (26-37)	9 (6-14)
4 (n = 462)	142.24 (92.8-221.03)	49.08 (35.0-72.0)	3 (2-3.8)	8.1 (7.9-8.3)	49 (44-55)	18 (12-25)	31 (25-37)	10 (7-13)
5 (n= 176)	162.34 (102.04-257.62)	57.35 (34.32-85.3)	3.05 (2.3-4)	8.1 (7.9-8.3)	48.5 (45-53)	20 (11-25)	31 (26-38)	10 (8-15)
6 (n = 40)	196.6 (111-54-286.1)	62.32 40.62-89.5)	3.05 (2.25-4.9)	7.9 (7.9-8.3)	48 (45-55)	20 (10.5-23.5)	30.5 (25.5-39.0)	10 (5.0-16.0)
7 (n = 29)	191.1 (133.5-319.2)	72 (40.0-88.0)	3.5 (2.5-4.0)	8.1 (7.9-8.3)	48 (45-54)	20 (12-22)	30 (25-36)	12.5 (9.0-17.5)
p-value^1^	<0.01	<0.01	<0.01	0.75	0.43	<0.01	<0.01	0.03

Data are expressed as median (IQR).

EA, ejaculation abstinence; IQR, interquartile range; TSC, total sperm count.

^1^ Not parametric trend analysis.

In the population of patients with sperm abnormality, we found a significant trend increase from day 1 to day 7 of TSC (16.38 vs. 56.0; p<0.01), sperm concentration (million/ml) (8.0 vs. 18.0; p<0.01) and morphology (3.0 vs. 5.0; p<0.01). Interestingly, we found a significant trend decrease of motility (A+B) (28.0% vs. 21.0%; p<0.01) and pH (8.1 vs. 7.9; p<0.01) (**Table 4**).

**Table 4:** Sperm parameters according to ejaculation abstinence (EA) in patients with sperm abnormalities.

EA	TSC (million)	Sperm concentration (million/ml)	Volume (ml)	pH	Motility A+B (%)	Motility C (%)	Motilità D (%)	Morphology (%)
1 (n = 341)	16.38 (3.75-36)	8 (2-18)	2 (1.4-3)	8.1 (7.9-8.5)	28 (20-37)	23 (20-30)	47 (40-55)	3 (3-8)
2 (n = 1,722)	20 (4.5-50)	10 (2-24)	2.3 (1.6-3)	8.1 (7.9-8.3)	30 (20-37)	23 (18-30)	45 (39-54)	4 (2-7)
3 (n = 4,038)	32.295 (5.6-94.05)	12.55 (2.2-34.1)	2.8 (2-3.8)	8.1 (7.9-8.3)	23 (11-33)	30 (20-39)	43 (34-55)	5 (2-8)
4 (n = 4,910)	42.27 (6.44-126)	15.4 (2.28-43.05)	3.1 (2.2-4.2)	8.1 (7.9-8.3)	22 (10-32)	31 (20-40)	42 (33-55)	5 (3-8)
5 (n = 1,898)	51.249 (7.03-145.75)	15.95 (2.1-46.75)	3.3 (2.3-4.5)	8.1 (7.9-8.3)	21 (8-32)	32 (21-39	42 (33-56)	5 (2-8)
6 (n = 337)	74.52 (11-197.4)	23.1 (4-57.2)	3.3 (2.1-4.6)	8.1 (7.9-8.3)	21 (10-30)	34 (23-41)	40 (31-51)	5 (3-8)
7 (n = 281)	56 (6.24-176)	18 (2.4-56.1)	3.4 (2.2-4.6)	7.9 (7.9-8.1)	21 (10-32)	24.5 (11.5-36)	47 (33.5-60)	5 (2-8)
p-value	0.01	0.01	0.01	0.01	0.01	0.01	0.43	0.02

Data are expressed as median (IQR).

EA, ejaculation abstinence; IQR, interquartile range; TSC, total sperm count.

1 Not parametric trend analysis.

In patients affected by asthenospermia, motility (A+B) dropped significantly from day 1 to day 7 (11.8% vs. 6.1%; p<0.01) and also in patients with teratospermia morphology dropped significantly (2.13% vs. 1.26%; p<0.01).

In the overall population, at the age adjusted logistic regression analysis, Q2 (EA between 3 and 4 days)(odds ratio [OR]: 1.52; 95%CI 1.32-1.75; p<0.01) and Q3 (EA ≥ 4 days) (OR: 1.45; 95%CI 1.21-1.73; p<0.01) were associate with OAT.

Similarly, Q2 (OR: 1.5281 95%CI 1.60-2.05; p<0.01) and Q3 (OR: 1.73; 95%CI 1.47-2.04; p<0.01) were associate with asthenospermia.

**Figure 1:**
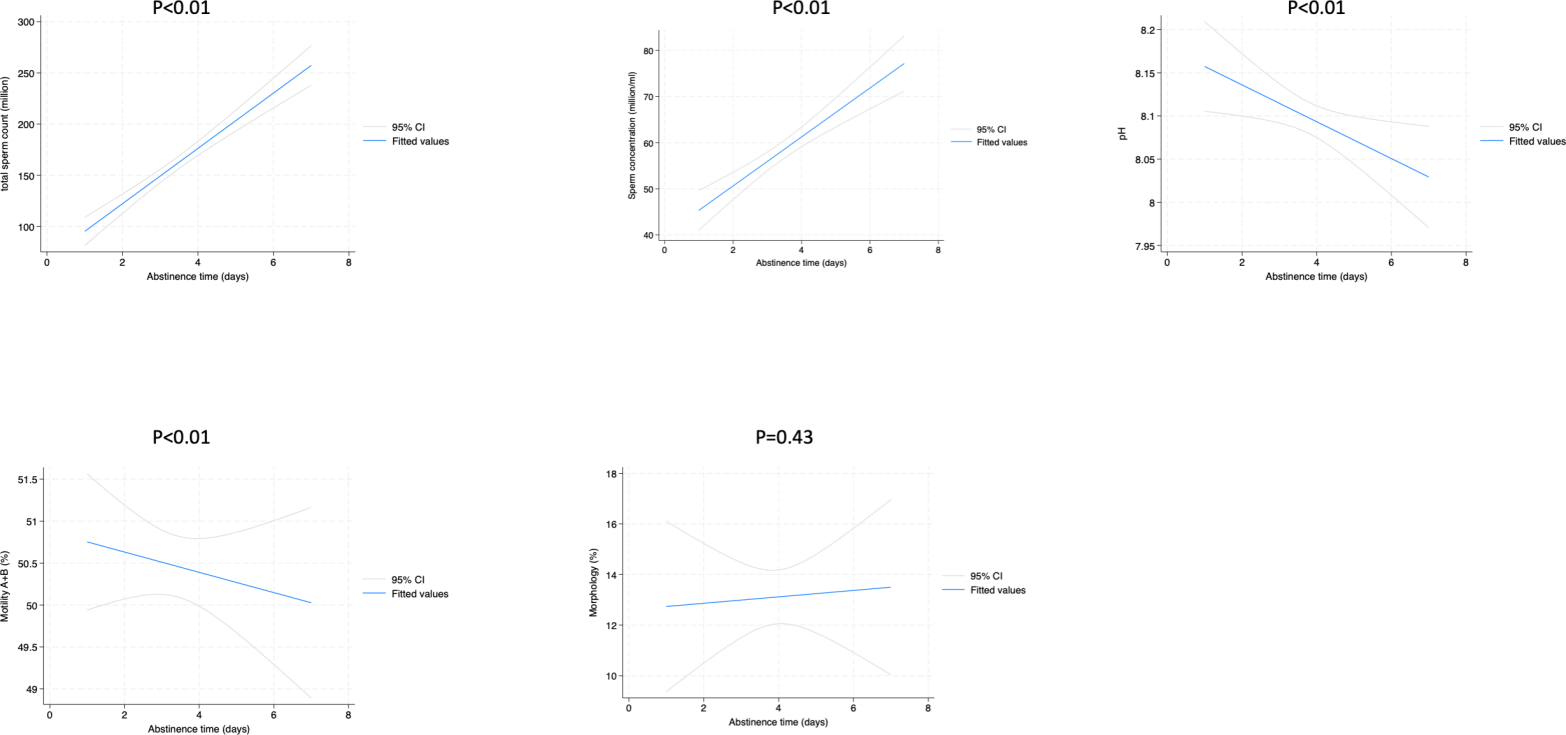
Linear regression plot between abstinence time and semen parameters in normospermic patients.

**Figure 2:**
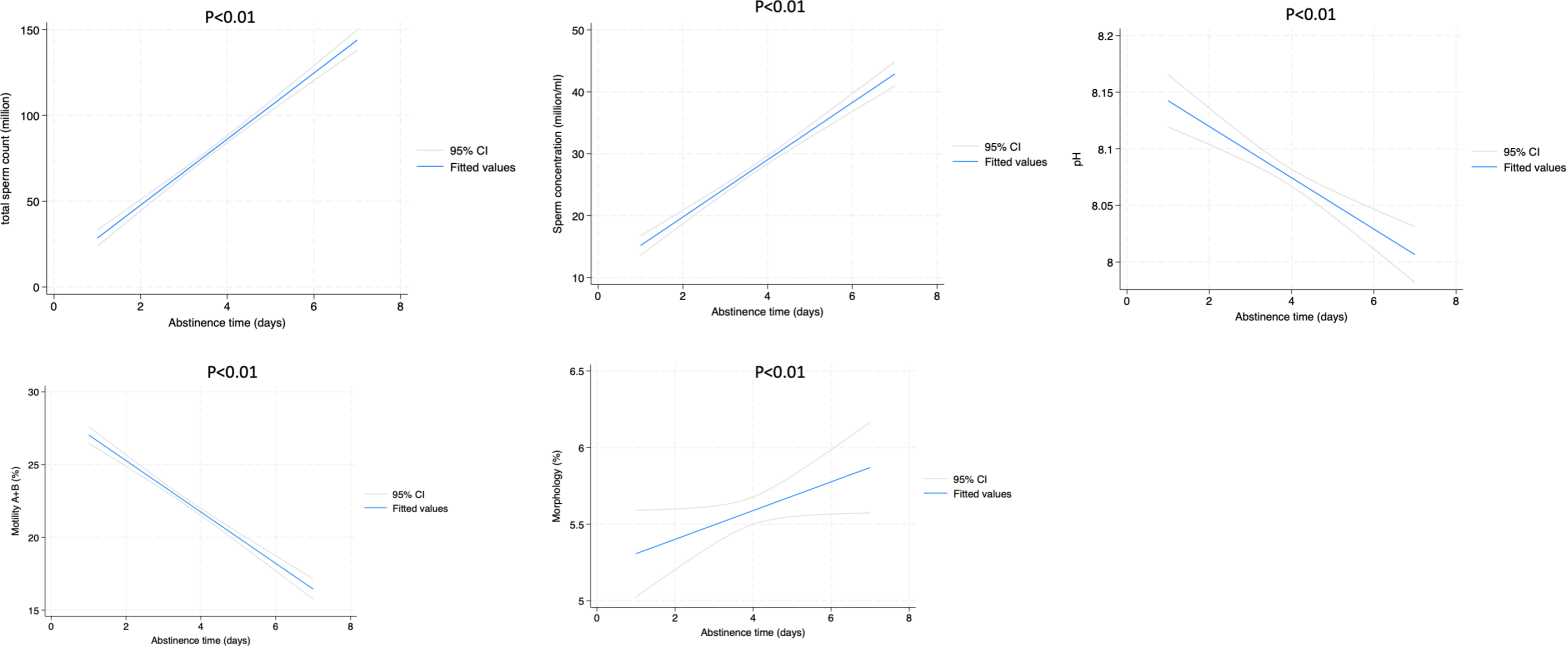
Linear regression plot between abstinence time and semen parameters in patients with sperm alterations.

**Figures 1**, **2** show the liner regression plot between abstinence time and semen parameters in normospermic and sperm abnormal patients.

## Discussion

Herein, we reported that abstinence time is a significant driver that can influence sperm parameters, in normal patients but even in patients with sperm abnormality.

Semen quality, encompassing parameters like sperm concentration, motility, and morphology, is a vital predictor of male fertility potential. In recent years, the role of abstinence duration in influencing semen quality has received increased attention. Current evidence suggests that a period of abstinence impacts semen parameters differently based on individual fertility profiles, distinguishing normospermic (normal semen quality) from sub-fertile men. This study builds upon these findings by examining a large cohort of 23,527 participants, offering valuable insights into how abstinence duration affects semen characteristics and potentially guides clinical practice in fertility treatments.

These results confirm existing evidence that sperm concentration and count are closely linked to fertility potential, as low sperm counts are consistently correlated with reduced chances of successful conception, but conventional semen analysis cannot reliably predict the chance of pregnancy or differentiate fertile vs. infertile men (except in the most extreme cases) (13).

Our study investigates how varying abstinence periods from 1 to 7 days affect semen parameters in normospermic and sub-fertile men. The findings highlight significant trends and provide insights into optimal abstinence durations for different fertility profiles.

In normospermic men, longer abstinence periods correlate with increased sperm concentration, TSC, and morphology. However, motility (A+B) remains relatively stable (49.8% to 49.9%; p = 0.43), indicating that while abstinence boosts sperm volume and quality, it does not enhance motility. These results align with research suggesting that sperm count and concentration benefit from abstinence due to the accumulation of spermatozoa in the epididymis, allowing for higher ejaculate volumes (14).

During transport and storage in the epididymis, sperm are exposed to elevated levels of reactive nitrogen and oxygen species (ROS), which can impair their quality (15). Additionally, incomplete emptying of the epididymis after a single ejaculation (16) allows residual sperm from previous ejaculations to affect the quality of subsequent samples.

Moreover, Yoshiakwa‐Terada et al. reported that patients with high SDF have significantly higher age and sexual abstinence duration and significantly lower forward progressive motility, compared to patients with low SDF (17).

A very recent meta-analysis demonstrated of 22 studies (31,640 samples) revealed that shorter abstinence significantly improved total and progressive motile sperm counts and reduced DNA fragmentation, though it decreased semen volume and sperm concentration. Oligospermia patients particularly benefited from improved sperm morphology and reduced DNA fragmentation (6).

Sánchez-Martín et al. further highlighted the pivotal role of recurrent ejaculation (REC) in improving sperm DNA integrity and enhancing reproductive outcomes in ICSI cycles. The significant reduction in sperm DNA fragmentation (SDF) observed following REC, compared to abstinence (ABS), emphasizes the potential of this approach to optimize sperm quality. Importantly, the improved pregnancy rate in the REC group (56.4%) versus the ABS group (43.3%) suggested that the lower SDF achieved through REC positively influences fertilization success. These results underscored the need to reconsider traditional abstinence protocols prior to ART and suggest that REC, coupled with density gradient centrifugation, may offer a simple yet effective strategy to maximize ICSI success rates (18).

Interestingly, the stability of motility in normospermic men suggests a potential ceiling effect, where additional sperm count does not contribute to increased movement. This could have implications for timing in assisted reproductive techniques (ART) like intrauterine insemination (IUI), where high motility is critical for success (19).

For patients with sperm alterations, abstinence similarly raises TSC, concentration, and morphology scores over 7 days. However, the data reveals a concerning trend with motility, which decreases significantly from 27.8% on day 1 to 20.8% by day 7 (p < 0.01). This reduction in motility with extended abstinence is particularly relevant, as motility is essential for sperm to navigate through the female reproductive tract. Reduced motility over time may be attributed to sperm aging and oxidative stress, which accumulate as sperm remain stored in the male reproductive tract (20).

To this regard, Jiang et al. demonstrated that penultimate ejaculatory abstinence adversely affects sperm DNA fragmentation, progressive motility, and sperm vitality, increasing the likelihood of asthenozoospermia, necrozoospermia, and elevated DNA fragmentation levels, highlighting the importance of seminal fluid stasis the accumulation of aging sperm, and the clearance of these sperm cells (21).

Moreover, asthenospermia and teratospermia display distinctive changes with abstinence. In asthenospermic men, motility (A+B) drops sharply from 11.8% on day 1 to 6.1% on day 7 (p < 0.01), while teratospermic patients show a significant decline in normal morphology from 2.13% to 1.26% (p < 0.01). These findings suggest that patients with already compromised motility or morphology may experience further declines with prolonged abstinence, supporting a personalized approach to fertility recommendations. Oxidative stress may further explain this motility decline, as reactive oxygen species (ROS) accumulate over time, leading to damage that particularly affects motility in asthenospermic individuals (22, 23).

The study’s findings underscore the need for individualized abstinence guidelines based on patient-specific semen profiles, especially for sub-fertile men and those with specific abnormalities like asthenospermia or teratospermia.

For normospermic men, a longer abstinence period of up to 7 days may be advantageous, as it increases sperm concentration and TSC without negatively impacting motility. This duration can be ideal for semen collection in natural conception and ART procedures that prioritize sperm count, such as IUI (10).

Sub-fertile men, particularly those with compromised motility or morphology, may benefit from shorter abstinence durations to avoid further reductions in motility. In patients with asthenospermia, an abstinence period of less than 4 days may help retain motility while still allowing for an adequate concentration and TSC for ART interventions. This approach aligns with studies recommending shorter intervals for men with poor motility or morphology, as longer durations exacerbate oxidative stress and sperm damage (9, 24).

The biological underpinnings of how abstinence impacts semen quality, particularly motility, in sub-fertile men are an area warranting further exploration. Oxidative stress, sperm aging, and DNA fragmentation are likely contributors, especially in cases of prolonged abstinence. Studies have shown that ROS damage the membrane integrity of spermatozoa, impairing motility and morphology and thereby lowering fertilization potential (22).

This study provides robust evidence highlighting the pivotal role of abstinence time in shaping semen quality across different male fertility profiles. This has critical implications for the timing of assisted reproductive techniques (ART) like intrauterine insemination (IUI), where motility plays a decisive role. For sub-fertile men, our findings reveal that prolonged abstinence can further compromise motility and morphology, particularly in cases of asthenospermia and teratospermia. The study’s contribution lies not only in its large-scale data but also in providing actionable insights for clinical practice. By delineating the distinct responses of normospermic and sub-fertile men to varying abstinence durations, this research underscores the necessity of a personalized approach to semen analysis and fertility treatments. Additionally, it bridges gaps in our understanding of oxidative stress and its impact on sperm quality, paving the way for future interventions such as antioxidant therapies and dietary modifications.

Our findings support a paradigm shift toward precision medicine in male fertility management. Future research should continue to explore the biological mechanisms underlying these observations and evaluate the efficacy of targeted therapies to mitigate the effects of oxidative stress and DNA fragmentation. By advancing our understanding of the interplay between abstinence and semen quality, this study contributes to optimizing fertility outcomes in both natural and assisted reproductive contexts.

## Conclusions

This study presents evidence that abstinence duration significantly impacts semen quality in both normospermic and patients with sperm alterations, with distinct implications for each group. Patients with sperm alterations, especially those with motility or morphology issues, may experience declines in key parameters with prolonged abstinence, supporting the use of shorter intervals for these patients. Even if in normospermic men longer abstinence may enhance sperm concentration and morphology, shorter abstinence durations could improve motility and reduce DNA fragmentation, both critical factors for successful fertilization. Therefore, abstinence strategies should be individualized, accounting for the specific fertility context and prioritizing parameters most relevant to reproductive outcomes.

These findings pave the way for more personalized fertility recommendations and underscore the need for individualized abstinence guidelines to optimize semen quality, thus potentially improving outcomes in natural conception and ART. Clinicians may consider using this data to tailor advice on abstinence periods based on each patient’s semen profile, thereby contributing to more effective fertility management. The findings underscore the importance of standardizing abstinence duration during sperm analysis to mitigate the variability introduced by differing abstinence intervals, especially in the context of fertility evaluation and management.

In summary, the retrospective analysis revealed distinct trends in semen parameters based on abstinence duration, emphasizing the need for careful consideration of ejaculatory abstinence in the interpretation of sperm analysis results and fertility assessments, particularly regarding the decrease in motility in patients with asthenospermia and abnormalities in morphology in patients with teratospermia.

## Data Availability

The raw data supporting the conclusions of this article will be made available by the authors, without undue reservation.
